# Quantitative evaluation of extended-spectrum β-lactamase-producing *Escherichia coli* strains in the wastewater of a French teaching hospital and relation to patient strain

**DOI:** 10.1186/s13756-016-0108-5

**Published:** 2016-03-29

**Authors:** Laurence Drieux, Sophie Haenn, Laurent Moulin, Vincent Jarlier

**Affiliations:** UPMC Univ Paris 06, EA 1541, Laboratoire de Bactériologie, F-75005 Paris, France; AP-HP, Hôpitaux Universitaires la Pitié Salpêtrière-Charles Foix, Laboratoire de Bactériologie-Hygiène, F-75013 Paris, France; Eau de Paris, 33 avenue Jean Jaurès, 94200 Ivry-sur-Seine, France; Faculté de médecine Pierre et Marie Curie – Site Pitié-Salpêtrière, 91 boulevard de l’hôpital, 75634 Paris, Cedex 13 France

**Keywords:** Wastewater, Lactamase, *Escherichia coli*, Quantification

## Abstract

**Background:**

Extended-spectrum β-lactamase-producing *Escherichia coli* has become ubiquitous and has been reported in diverse ecosystems. We evaluated the potential impact of post-acute and long-term healthcare activities on the environment by quantifying ESBL-producing Enterobacteriaceae in wastewaters of a French geriatric hospital.

**Methods:**

We collected wastewater specimens representative of one-day efflux immediately before the connection with the municipal sewer pipe. The sample was processed following two different methods: dilution-filtration method and concentration method and was screened for ESBL-producing Enterobacteriaceae using selective media. ESBL *E. coli* strains were quantified, screened for ESBL genes and compared with ESBL strains isolated from patients present in the building at the time of wastewater collection, using molecular methods.

**Results:**

Six distinct environmental ESBL *E. coli* clusters were identified, two of them related to patient strains. The concentrations in hospital wastewater of these strains ranged from 2.5 × 10^4^ to 10^6^ UFC/L.

**Conclusions:**

Our results demonstrate that the presence of ESBL *E. coli* patients leads to a dissemination of ESBL *E. coli* in the environment and highlights the need to improve excreta and wastewater policy in hospitals.

## Background

The first extended-spectrum β-lactamases (ESBLs) were isolated in the 1980’s [1, 2]. Since the early 2000’s, CTX-M-type ESBLs have spread worldwide among *Escherichia coli* strains in hospitals and in the community [3, 4]. ESBL-producing *E. coli* have been reported in diverse human communities, even in remote populations [5], pets [6, 7], farm animals [8–10], lakes and river [11], wildlife [12] and soils [10]. Presence of ESBL-producing *E. coli* has been demonstrated in hospital wastewater [13–17] however the link between the presence of ESBL-producing *E. coli* in hospital wastewater and patients infected or colonized with such bacteria remains unclear. In this study, we focused on a building of our hospital and quantified ESBL-producing Enterobacteriaceae in wastewaters discharged from this building.

## Methods

### Hospital setting

The study was conducted at the Charles Foix hospital, a 450-bed teaching medical institution located at Ivry-sur-Seine, suburb of Paris, France. We focused the study on the building Calmette et Guérin (CG, 126 beds) which is constituted of geriatric post-acute care (PAC), long-term care (LTC) units along with a little psychiatric unit.

### Wastewater collection

From December 29^th^ 2010 9.30 am until December 30^th^ 2010 9.30 am, wastewater samples were collected at the sewer pipe that collects wastewaters from the CG building, immediately before the connection with the municipal sewer pipe. At this collection point, 140-mL samples were collected every 15 min within 24 h using an automatic refrigerated water sampler (Teledyne Isco, Lincoln, NE, USA). These samples were mixed to constitute a specimen representative of one-day efflux. Flow, pH and temperature were continuously monitored.

### Wastewater processing

The sample was processed on the same day following two methods, i.e. the dilution-filtration method and the concentration method.

In the dilution-filtration method, the sample was diluted in sterile saline solution (10^-2–7^) and 2 volumes of 100 mL of each diluted suspensions were filtrated on 0.45 μm nitrocellulose membranes (Merck Millipore, Billerica, MA, USA). These membranes were deposited on two distinct selective agars: chromID® ESBL agar (bioMérieux, Marcy l’Etoile, France) and Drigalski agar supplemented with cefotaxime (0.5 mg/L) and ticarcillin (250 mg/L) (Drig-TC).

In the concentration method, 20 mL of the wastewater specimen were filtrated on a 0.4 μm polycarbonate Isopore® membrane (Merck Millipore, Billerica, MA, USA) and then suspended in 5 mL sterile saline solution using a sterile cell scraper. These suspensions were then diluted in sterile saline solution (10^-1–6^) and 100 μL of each diluted suspension were plated on both chromID® ESBL and Drig-TC agars.

### Patients

The microbiological database was screened for patients present in the building at the time of wastewater collection and whose bacteriological specimens, taken either for clinical or bacteriological (i.e. rectal swab) were positive for ESBL-producing *E. coli*.

### Bacterial strains and susceptibility assays

Environmental, epidemiological and clinical strains were identified by API 20E® system (bioMérieux, Marcy l’Etoile, France). Susceptibility to antimicrobial agents was assessed by disk diffusion method according to French guidelines (www.sfm-microbiologie.org) and ESBL production was screened by the double disk synergy test (DDST) [18].

### Beta-lactamase characterization and screening of resistance genes

Each strain displaying a positive DDST was screened for the presence of for the *bla*_TEM,_*bla*_SHV_ and *bla*_CTX-M_ genes, using PCR amplification and sequencing as reported before [19].

### Molecular typing

Wastewater and patient ESBL-producing strains of *E. coli* were compared using the semi-automated typing system DiversiLab® (bioMérieux, Marcy l’Etoile, France) as described elsewhere [20] using 95 % similarity threshold to consider fingerprints as similar.

When DiversiLab fingerprints were considered as similar, the corresponding strains were further studied by Pulsed-Field Gel Electrophoresis (PFGE) as reported before [21].

## Results

At collection point, the flow was 39,270 m^3^ per day, the temperature ranged from 12 to 17.3 °C and the pH ranged from 6.2 to 8.9.

Each distinct colony that grew on selective agar plates (chromID® ESBL and Drig-TC) inoculated with wastewater processed by dilution-filtration or concentration method was identified and screened for ESBL production. A total of 389 colonies could be individualized and were studied. Most of them were non fermentative bacteria or cephalosporinase-producing enterobacteria. Twenty-five colonies were identified as ESBL-producing Enterobacteriaceae, including 23 *E. coli* and two *Enterobacter cloacae*. The concentrations of ESBL-producing *E. coli* in wastewater was estimated using the number of colonies, the dilution factor and the volume filtrated. This estimated concentrations ranged between 1 × 10^6^ UFC/L based on the characteristics of the dilution-filtration method, and 2.5 × 10^5^ UFC/L based on the characteristics of the concentration method.

Nine patients hospitalized in the CG building at the time of wastewater collection had been identified carrying ESBL *E. coli*. The 23 isolates from wastewater and 9 strains isolated from 9 carriers were submitted to genotyping and identification of ESBL-encoding genes.

The 23 wastewater ESBL *E. coli* isolates belonged to 5 distinct DiversiLab® patterns: 14 to pattern 1, four to pattern 2, two to patterns 3 and 5, respectively, and one to pattern 4 (Table 1). The isolates belonging to the DiversiLab® profile 1 was subdivided into two pulsotypes 1a and 1b. PFGE 1a corresponded to isolates carrying the ESBL *bla*_CTXM-14_ and the broad-spectrum β-lactamase *bla*_TEM-1_ genes. PFGE 1b corresponded to ESBL CTX-M-27-producing isolates (Table 1). Other clusters produced CTX-M-1 or TEM-3.

**Table 1:** Characteristics of the ESBL-producing *Escherichia coli* clusters isolated from wastewater specimen

Cluster	Range of enumeration in wastewater (UFC/L) ^a^	β-lactamases	DiversiLab® pattern	Pulsotype
WWCG1a	2 × 10^4^ – 4 × 10^4 a^	CTX-M-14, TEM-1	1	1a
WWCG1b	2.5 × 10^4^ – 2.5 × 10^5 a^	CTX-M-27	1	1b
WWCG2	1 × 10^4^ - 1 × 10^6 a^	CTX-M-1	2	-
WWCG3	2 × 10^4^	TEM-3	3	-
WWCG4	1 × 10^4^	TEM-3	4	-
WWCG5	2 × 10^4^	CTX-M-1, TEM-1	5	-

^a^Concentration estimated for both the dilution-filtration method and the concentration method

Thus, a total of six distinct clusters of ESBL-producing *E. coli* clusters (WWCG1a to WWCG5) were identified in wastewater. The concentrations of each cluster was estimated using the number of colonies belonging to this cluster, the dilution factor and the volume filtrated. The estimated concentration ranged from 2.5 × 10^4^ to 10^6^ UFC/L (Table 1). Most of them (4/6) produced a CTX-M-type ESBL, the others producing TEM-3.

The genotyping results obtained for the 9 ESBL-producing *E. coli* strains from patients, showed that five of them were undistinguishable (Table 2). These five strains were also undistinguishable from the wastewater WWCG1b cluster defined above and carried the *bla*_CTX-M-27_ gene. These strains had been isolated from three patients hospitalized in the same post-acute care ward (PAC-Mong) (Table 2), from one patient hospitalized in the long-term care ward but who had been previously hospitalized in PAC-Mong unit at the same time as the above patients, and from one patient hospitalized in a distinct post-acute care ward (PAC-Nec) that shares nursing and medical staffs with PAC-Mong ward. One patient strain cluster belonged to DiversiLab® profile 1 and pulsotype 1a, carried *bla*_CTXM-14_ and *bla*_TEM-1_ genes and consequently was undistinguishable from wastewater cluster WWCG1a (Table 2).

**Table 2:** Characteristics of the ESBL-producing *Escherichia coli* strains isolated from patients present at the time of wastewater collection

Patient	Unit of hospitalization^a^	Specimen	β-lactamases	DiversiLab® type	Pulsotype	Corresponding wastewater cluster^b^
1	PAC-Nec.	Rectal swab	CTX-M-27	1	1b	WWCG1b
2	PAC-Mong.	Rectal swab	CTX-M-27	1	1b	WWCG1b
3	PAC-Mong.	Rectal swab	CTX-M-27	1	1b	WWCG1b
4	PAC-Mong.	Rectal swab, urine	CTX-M-27	1	1b	WWCG1b
5	LTC	wound	CTX-M-27	1	1b	WWCG1b
6	PAC-UPG	Urine	CTX-M-14, TEM-1	1	1a	WWCG1a
7	PAC-Mong.	Rectal swab	CTX-M-15, TEM-1	1	1c	-
8	LTC	Rectal swab	CTX-M-1, TEM-1	6	-	-
9	PAC-Mong.	Rectal swab	CTX-M-15, TEM-30	7	-	-

^a^unit of hospitalization at the time of wastewater collection; *PAC* post-acute care wards, *LTC* long-term care ward; ^b^see Table 1 for definition

The other three patients strains were distinct one from each other and also distinct from wastewater isolates.

## Discussion

Enterobacteriaceae, particularly *E. coli*, are commensal bacteria present at about 10^8^ colony-forming units per gram of human faeces. The faecal concentration of ESBL-producing *E. coli* may vary with the antibiotic treatment and it has been shown that the mean relative faecal abundance of ESBL-producing *E. coli* was 13-fold higher in women exposed to antimicrobials [22]. Even before ESBLs were first reported, hospitals were considered as sources of environmental pollution with multiresistant *E. coli* strains. Indeed, in 1973, Grabow et al. reported that the proportion of *E. coli* carrying multiresistant conjugative plasmids was higher (26 % *vs* 4 %) in hospital wastewater samples than in city sewage samples [23]. As hospital settings concentrate ESBL-producing *E. coli* carriers, they can be a source of environmental ESBL-type pollution.

Several studies have focused on hospital discharge of ESBL-producing Enterobacteriaceae [13–17]. However, in most cases, sewages were sampled by filling directly sterile bottles, i.e. by studying random specimens of wastewater. In our study, the sewage point was sampled using equipment allowing the collection of a specimen representative of a daily wastewater discharge, taking into account quantitative and qualitative variations of flow that can be observed within a single day.

Moreover, in most of the above studies, Enterobacteriaceae were enumerated but the presence of ESBL-producing strains was notified without the range quantification [13–16]. In our study, we estimated the concentration of ESBL-producing *E. coli* based on the specimen processing. Concentrations we have found are consistent with the concentration of cefotaxime-resistant *E. coli* strains reported by Galvin et al. in the effluents of an Irish hospital (estimation, 10^5^ UFC/L) [15]. Most of the environmental ESBL *E. coli* isolates (20/23) found in our hospital wastewater produced a CTX-M-type ESBL belonging either to group 1 or to group 9 and few to TEM type. This predominance of CTX-M-type ESBLs is consistent with the relative distribution of ESBL enzymes among clinical ESBL *E. coli* in France [24].

Molecular typing of wastewater ESBL isolates allowed identifying and quantifying two clusters carried by patients present in the building at the time of wastewater collection.

To our knowledge, the present study is the first report of ESBL-producing *E. coli* quantification in sewage in direct relation with strains isolated from patients.

## Conclusions

The present study demonstrates that the presence of ESBL-producing *E. coli* carriers in hospital may lead to a dissemination of ESBL-producing *E. coli* in the environment, with a quantification of ESBL-producing *E. coli* in sewage in direct relation with strains isolated from patients. In this matter, Healthcare settings play an important role in ESBL-producing bacteria spread, not only through cross-transmission and antibiotics consumption, but also through their wastes and should improve their disposal of human excreta in order to reduce their impact on the environment.

## Abbreviations

DDST: double disk synergy test
Drig-TC: Drigalski agar supplemented with cefotaxime and ticarcillin
ESBL: extended-spectrum β-lactamase
ESBL-E: extended-spectrum β-lactamase-producing Enterobacteriaceae
LTC: long-term care
MRSA: methicillin-resistant *Staphylococcus aureus*
PAC: post-acute care
PCR: polymerase chain reaction
PFGE: pulsed-field gel electrophoresis
UFC: unit forming colony
WWTP: wastewater treatment plant
